# A review of lightweight convolutional neural networks for ultrasound signal classification

**DOI:** 10.3389/fphys.2025.1536542

**Published:** 2025-04-24

**Authors:** Bokun Zhang, Zhengping Li, Yuwen Hao, Lijun Wang, Xiaoxue Li, Yuan Yao

**Affiliations:** ^1^ School of Information Science and Technology, North China University of Technology, Beijing, China; ^2^ Disaster Medicine Research Center, Medical Innovation Research Division of the Chinese PLA General Hospital Beijing, China Beijing Key Laboratory of Disaster Medicine, Beijing, China; ^3^ Hangzhou Institute of Technology, Xidian University, Xi’an, China; ^4^ Emergency Department, 903rd Hospital of PLA Joint Logistic Support Force, Hangzhou, China

**Keywords:** ultrasound, signal classification, lightweight technology, model compression, optimization of lightweight network, convolutional neural network

## Abstract

Ultrasound signal processing plays an important role in medical image analysis. Embedded ultrasonography systems with low power consumption and high portability are suitable for disaster rescue, but due to the difficulty of ultrasonic signal recognition, operators need to have strong professional knowledge, and it is not easy to deploy ultrasonography systems in areas with relatively weak infrastructures. In recent years, with the continuous development in the field of deep learning and artificial intelligence, lightweight convolutional neural networks have brought new opportunities for ultrasound signal processing. This paper focuses on investigating lightweight convolutional neural networks applied to ultrasound signal classification. Combined with the characteristics of ultrasound signals, this paper provides a detailed review of lightweight algorithms from two perspectives: model compression and operational optimization. Among them, model compression deals with the overall framework to reduce network redundancy, and the latter aims at the lightweight design of the basic operational module “convolution” in the network. The experimental results of some classical models and algorithms on the ImageNet dataset are summarized. Through the comprehensive analysis, we present some problems and provide an outlook on the future development of lightweight techniques for ultrasound signal classification.

## 1 Introduction

Ultrasound imaging is a crucial medical imaging technology that, compared to CT and X-ray, offers portability, simplicity, and no ionizing radiation, making it ideal for deployment in resource-limited environments such as disaster relief. However, ultrasound images are often complex and susceptible to noise, requiring doctors to rely on subjective experience for diagnosis. Integrating artificial intelligence can assist in recognition, but traditional deep convolutional neural networks (Krizhevsky et al., 2012; Simonyan and Zisserman, 2014; Szegedy et al., 2015; He et al., 2016), with high computational demands and large parameter sizes, are unsuitable for portable ultrasound devices. Lightweight models reduce computational requirements, enabling real-time ultrasound image processing to help doctors diagnose conditions more quickly and accurately, reducing patient wait times (Liu et al., 2023). Additionally, lightweight models minimize dependence on specialized skills and complex equipment, improving the accessibility and portability of ultrasound-based diagnostics. Given its lower cost compared to CT and X-ray, ultrasound imaging facilitates the widespread adoption of intelligent diagnostic technologies, particularly in developing countries and remote areas (Gooßen et al., 2019). Therefore, the popularization of lightweight technology in the ultrasound examination process can promote intelligent progress in medical image analysis, which can provide support for medical diagnosis and has significant application value and social significance.

In 2016, the first lightweight model SqueezeNet (Iandola, 2016) was made public to achieve results approximating AlexNet on the ImageNet dataset but with 1/50th of the model size of AlexNet. Song Han and Hinton proposed weight pruning (Han et al., 2015) and knowledge distillation (Hinton, 2015) respectively to reduce redundancy in deep network structure from the perspective of model compression. Lightweight techniques, mainly lightweight model construction and model compression, have triggered a large number of influential research and breakthroughs (Chen et al., 2023).

**FIGURE 1:**
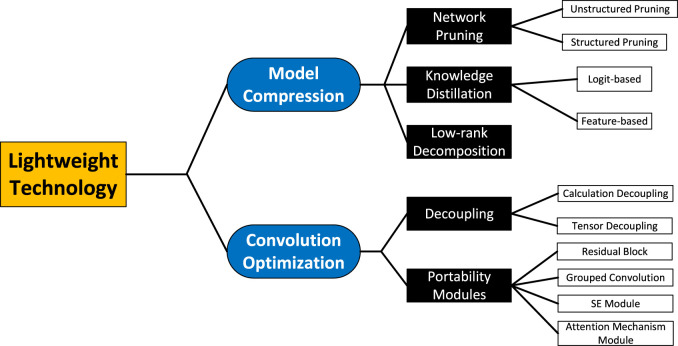
Classification of lightweight methods.

In the process of developing traditional neural networks, there may be a large amount of redundancy as the depth of the network deepens. This redundancy mainly consists of computational complexity and many parameters. To lighten the network and remove the redundancy, we need to optimize the model itself and the underlying framework, of which the basic modular unit of the underlying framework is “convolution”. Therefore, we divide the lightweight techniques into two directions: model compression and computational optimization. The former is to compress a large neural network into a lightweight network, mainly including network pruning, knowledge distillation and low-rank decomposition. The latter is to lighten the design of “convolution”, which is an operational module in the network. The lightweight technique can realize efficient signal analysis in resource-constrained environments. The classification of lightweight technologies is shown in Figure 1.

Artificial intelligence and computer-aided diagnostic solutions can significantly standardize medical practice, reduce training time, and improve the quality of ultrasound signals. There are four main research areas.

1. The invocation of machine learning techniques is expected to significantly improve signal quality and make imaging clearer (Deffieux et al., 2021). Emerging methods such as beamforming, super-resolution and data enhancement techniques have achieved some results (Micucci and Iula, 2022), but they often require hardware tuning. Despite the difficulty of implementation, research has gradually overcome the limitations of traditional image reconstruction algorithms, especially in the translation of ultrasound physical measurements into visualized images.
2. Artificial intelligence algorithms can help healthcare professionals perform a thorough examination, thus helping to reduce the learning curve of ultrasound scanning (Mischi et al., 2020)
3. The most competitive solutions currently available are deep learning-based image processing methods compared to traditional feature engineering methods (Sashidhar et al., 2021). These algorithms show significant advantages in measurement, quantification and computer-aided detection.
4. The application of computer-aided techniques in diagnosis and triage is receiving research attention because these methods can effectively reduce the burden on physicians and improve their efficiency (Van Sloun et al., 2019).

Ultrasound imaging quality is heavily operator-dependent, posing challenges for inexperienced practitioners. Over-filtering and improper gain adjustments, while improving texture smoothness, often reduce the clinical utility of images. Research into computer-aided scanning techniques aims to enhance automation, making ultrasound acquisition more efficient and accessible.

For developing countries, the impact of computer-aided scanning may be even more significant. High-quality ultrasound is inherently costly, many areas with poor infrastructure are not equipped for ultrasound (Gooßen et al., 2019). Also, the lack of experienced sonographers in developing countries prevents patients from undergoing timely ultrasound diagnosis such as prenatal examinations (Mischi et al., 2020). Therefore, the creation of a computer-aided scanning system could make it possible to perform ultrasound examinations in remote areas with people with only basic anatomical knowledge. Such a system could filter out images of clinical value and send them to radiologists for specialized diagnosis, even if they are thousands of miles away.

Despite its advantages, ultrasound imaging presents unique challenges due to its susceptibility to noise, variable feature scales, and complex temporal characteristics. These signal features not only affect the clarity and accuracy of imaging but also increase the computational burden of deep learning models in recognition and classification. Lightweight convolutional neural networks provide an effective solution for ultrasound signal classification, which achieves efficient signal analysis in resource-limited environments through model compression and computational optimization.

Ultrasound imaging can be divided into A-type, B-type and M-type ultrasound. A-type ultrasound displays the intensity of a single echo, the B-type ultrasound converts the A-type signals into two-dimensional ones for static analysis, and the M-type ultrasound imaging is simpler and does not require complex reconstruction, making it the first choice for portable ultrasound detection equipment. For M-typesignals, periodic signals show stable fluctuations, which is conducive to quantitative evaluation, while non-periodic signals clearly show abnormal features.

**FIGURE 2:**
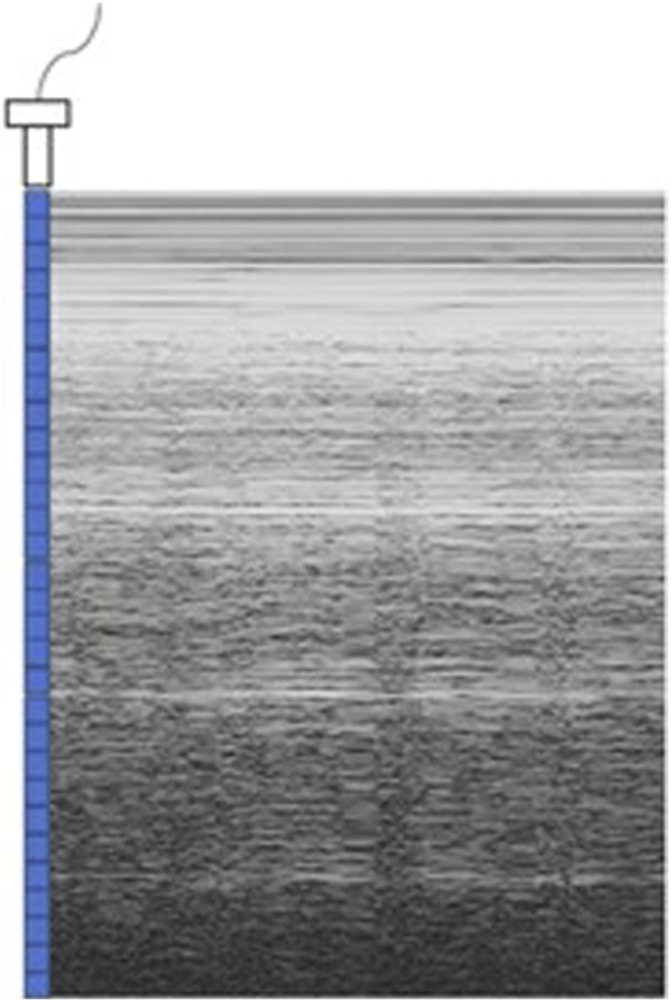
M-type ultrasound image.

**FIGURE 3:**
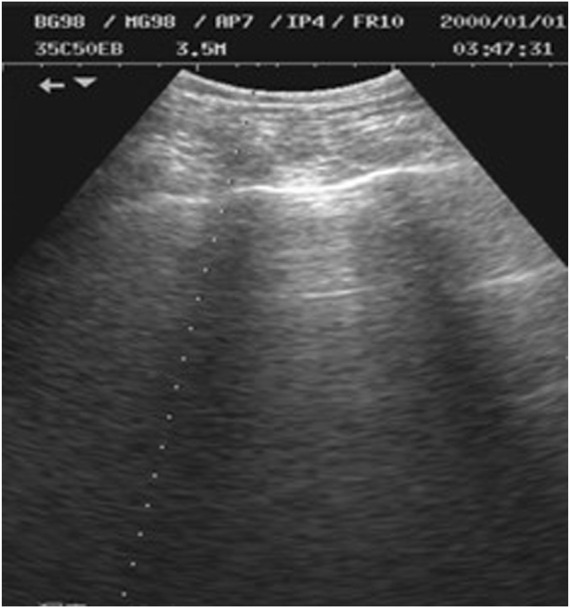
B-mode ultrasound rib image.

As shown in Figure 2, M-ultrasound measures the change of reflection intensity with depth and time along a fixed direction, with the horizontal coordinate indicating time, the vertical coordinate indicating depth information, and the brightness indicating the strength of the reflected signals. M-Ultrasound has a very high temporal resolution and is able to accurately capture the rapid movement of tissues or organs, which is very suitable for detecting dynamic tissues and organs. However, due to this dynamic characteristic, M-Ultrasound is more sensitive to images with scattering noise (Kang et al., 2015) and acoustic shadow effect (Matsuyama et al., 2022). Scattering noise is a type of grain-mounted noise in ultrasound imaging due to the coherent superposition of acoustic waves with scattering in tissues, which reduces the contrast and detail resolution of the image. The acoustic impedance difference between the bone and muscle tissues of the human body is extremely large, and after the ultrasound wave propagates through the body to the bone, the ultrasound will undergo total reflection at the bone interface due to the large acoustic impedance difference (Li, 2022), so the ultrasound wave cannot reach the posterior region. This phenomenon is known as the acoustic shadow effect. Figure 3 shows an image of the rib cage under B-mode ultrasound. The randomness and non-Gaussian distribution of the scattering noise make ultrasound denoising an important problem.

Therefore, for ultrasound signals, if the model focuses too much on higher-order feature correlations might enhance irrelevant noise patterns rather than improving target clarity. Ansari et al. (2023) annotated the extracted images using the Computer Vision Annotation Tool (CVAT). To enhance the contrast and highlight the liver boundary, the images were also preprocessed using Contrast Limited Adaptive Histogram Equalization (CLAHE) Reza (2004). Afsa et al. (2024) used uses Independent Component Analysis (ICA) to eliminate feature redundancy, extract independent components, and improve computational efficiency and model effect. This method can still maintain high prediction performance in the case of data imbalance. Regaya et al. (2023) effectively reduced image noise and enhanced feature expression by combining maximal overlap discrete wavelet transform (MODWT) and stochastic resonance (SR) (Dakua et al., 2019) technology in the preprocessing stage, providing high-quality input data for subsequent cerebral aneurysm segmentation tasks. Ansari et al. (2024) reviewed in detail preprocessing methods such as data enhancement and denoising for ultrasound signals, which effectively solved data scarcity and image quality problems and provided the possibility of building an end-to-end deep learning system.

The application of lightweight technology greatly reduces the computing resource requirements for ultrasound signal classification, making real-time ultrasound analysis possible. However, due to the high temporal resolution of ultrasound signals (such as M-mode ultrasound), the model is required to quickly process time series data to provide immediate feedback. Therefore, parallel computing technologies, such as GPU parallel processing and field programmable gate arrays (FPGAs), play a key role in real-time ultrasound feedback. Zhai et al. (2019a) proposed a hardware architecture based on Zynq SoC to accelerate the calculation of Lattice Boltzmann (LB) method (Mazzeo and Coveney, 2008). LB can be efficiently implemented on a variety of parallel architectures, ranging from general purpose graphics processing units (GPGPU) (Kuznik et al., 2010) and supercomputers (Djelouat et al., 2018). Based on the above research, (Zhai et al., 2019b) optimized the HemeLB model, designed an acceleration solution based on Zynq SoC and GPU, and proposed a real-time visualization framework, providing an efficient, scalable, and user-friendly tool for clinical application of hemodynamic simulation. Esfahani et al. (2020) proposed an integrated pipeline for cerebral aneurysm blood flow simulation and real-time visualization. This pipeline provides an efficient clinical tool for cerebral aneurysm blood flow simulation and visualization by combining GPU-accelerated HemeLB and a real-time rendering engine.

Through parallel computing, convolution operations, feature extraction, and signal classification can be performed simultaneously, thereby reducing latency and improving diagnostic efficiency. In addition, parallel computing can also be combined with deep learning technologies, such as the self-attention mechanism in the Transformer architecture, to effectively improve the processing capabilities of ultrasound time series signals and provide stronger intelligent auxiliary support for portable ultrasound devices.

## 2 Model compression

Model compression refers to the compression of model volume to achieve similar accuracy as the original model by reducing the model size, removing over-parameterization redundancy and structural redundancy, and reducing the memory footprint. Model compression based on computer-aided diagnostic techniques can further enhance the flexibility and deployment capability of ultrasound diagnostic systems. According to different processing ideas, model compression techniques can be mainly classified into network pruning, knowledge distillation and low-rank decompossion. Table 1 briefly summarises the characteristics of the three basic types of model compression methods.

**TABLE 1:** The comparison of basic methods for model compression.

Method	Applicable layers	Description	Advantages and disadvantages
Network Pruning	Convolutional layer and fully connected layer	Removing non-essential redundancies from the pre-trained model while maintaining accuracy	Enhances generalization and reduces overfitting risk but requires specialized libraries and hardware
Knowledge Distillation	Convolutional layer and fully connected layer	Allowing the student model to match the teacher model’s performance with lower computational and memory costs	Speeds up training and enhances performance but is limited to teacher-student setups
Low-rank Decomposition	Convolutional layer and fully connected layer	Decomposing convolution kernels to reduce redundancy	Improves computational efficiency; but hard to implement and decomposition operation requires a lot of computation

### 2.1 Network pruning

**FIGURE 4:**
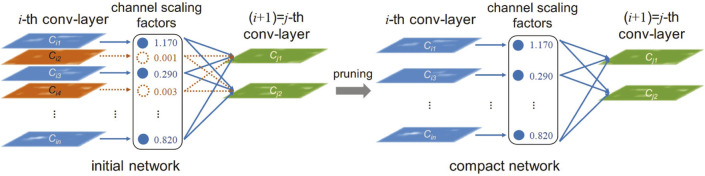
Network pruning.

In recent years, network pruning has been widely studied as a technique to reduce the computational and storage requirements of neural networks, especially for the compression of deep networks. Network pruning is used to eliminate non-critical redundancies in the pre-trained model without affecting the accuracy of the model. The process of network pruning is shown in Figure 4, where a scaling factor is assigned to each channel of the convolutional layer (Lai et al., 2018). During the training process, these scaling factors are constrained by sparse regularization to automatically identify unimportant channels. After constraints, channels with smaller scaling factors are pruned. After pruning, the model structure becomes more compact.

Network pruning techniques are widely used in ultrasound image segmentation and classification tasks, where the computational requirements of the model are reduced by pruning. M-ultrasound dynamically tracks data from only a single scan line, which has less data compared to B-ultrasound and 3D-ultrasound but requires a higher temporal resolution. The network pruning technique removes redundant convolutional kernels or channels and is suitable for M-Ultrasound. The pruned network is able to preserve dynamic signal features while reducing computational requirements (Liu et al., 2017). Usually, network pruning can be categorized into unstructured pruning and structured pruning by granularity.

#### 2.1.1 Untructured prunning

Unstructured pruning removes individual weights from the model, which minimizes the number of parameters and is commonly used for fine-grained pruning.

Song Han proposed the concept of Deep Compression in 2016 in order to solve the problem that neural networks are computationally and memory intensive and difficult to deploy on systems with limited hardware resources (He et al., 2016). It consists of three main stages: pruning, trained quantization, and Huffman coding. This deep compression concept compresses the neural network without compromising accuracy. Experiments on AlexNet (Krizhevsky et al., 2012), VGG-16 (Simonyan and Zisserman, 2014) and LeNet (Filtersâ€™Importance, 2016) networks were compressed by a factor of 35, 49, and 39, respectively, without loss of accuracy. After pruning, complex neural networks can be used in mobile applications with limited application size and download bandwidth.

However, magnitude-based weight pruning reduces a large number of parameters in the fully connected layer and may not sufficiently reduce the computational cost of the model due to irregular sparsity in the pruned network. Filtersâ€ ™Importance (2016) proposed to remove filters that have less impact on the accuracy of the output. Unlike pruning weights, this method does not produce sparse connection patterns.

Network pruning can also be combined with other model compression techniques. Park and No (2022) proposed a ‘prune-then-distill framework’, where the teacher model is first pruned to make it transferable and then distilled into the student model. Experiments have shown that distillation of the pruned teacher model can outperform the unpruned teacher model, which reverses the assumption that unpruned teacher networks are always more effective.

As research progressed, network pruning did not focus just on a single weight and certain modules of the neural network, but aimed at an entire layer of the network. Liao et al. (2023) investigated an EGP entropy-guided pruning algorithm, which targets layers in the network with low entropy values and prioritizes pruning their connections, eventually removing them completely. Through validation in popular models such as ResNet-18 and Swin-T (Liu et al., 2021), the EGP algorithm significantly compresses the depth of the model. The study reveals that unstructured pruning can also reduce the model depth.

Unstructured pruning has a promising application in ultrasound signal classification tasks. In particularly, it can significantly improve the computational efficiency and resource adaptability of the model when dealing with MMA image signals and time-domain signals. Experiments have shown that sparse networks can enhance the robustness of small-scale signals and can improve the accuracy of ultrasound signal classification (Srinivas et al., 2017). Meanwhile, unstructured pruning can be used to reduce unimportant temporal correlation weights, thus strengthening the model’s focus on key temporal features (Ayle et al., 2022), and improving the ability to classify heart rate or valve motion signals. In ultrasound time-domain signals, unstructured pruning can accurately remove low-contributing weights by weighted sparsity constraints in a specific time range, thus more efficiently processing signal parts with several energy species.

Unstructured pruning is promising for research due to its higher pruning rate and its ability to be combined with other model optimization techniques. In the future, unstructured pruning may combine dynamic pruning and sparse training techniques more often, allowing unstructured pruning methods to improve sparsity while enhancing hardware adaptability, thus speeding up inference.

#### 2.1.2 Structured pruning

Structured Pruning removes entire neurons, convolutional kernels, channels, or layers from a neural net. This type of pruning is easier to accelerate and is suitable for standard hardware platforms such as CPUs and GPUs.

In 2018, Yu et al. (2018) proposed the Neuron Importance Score Propagation (NISP) algorithm, where NISP assigns an importance score to each neuron to measure its overall contribution to network performance. By calculating the sensitivity of the network output to each neuron, pruning decisions can be made at the neuron level. NISP can keep neurons that contribute significantly to the final prediction in each layer, removing the least important neurons in the neural network. For ultrasound signal processing tasks, pruning out redundant channels and neurons can result in loss of high-frequency details in the ultrasound image, affecting edge clarity, especially in small lesion detection tasks (Bria et al., 2020). In contrast, this structured pruning prioritizes the retention of the extraction layer of key features and reduces the damage to high-frequency details (Wang et al., 2021).

Structured pruning usually imposes sparse constraints on the weight parameters and prunes some unimportant weights during the training process. Shao and Shin (2022) proposed a dynamic scheme that imposes sparse constraints based on the filter weights. This method evaluates the structure of the model by its performance in real-time and dynamically prunes it according to the current performance. Wen et al. (2016) proposed the structural sparsity learning (SLL) method to regularize the filters, channels, and layer depths in neural networks. This approach allows deep neural networks (DNNs) to learn more compact structures without loss of accuracy. The compactness of DNNs speeds up DNN evaluation on CPUs and GPUs using off-the-shelf libraries.

Structured pruning requires modifying the network architecture and implementing complex gradient update rules will offset some of the efficiency gains. For some more complex and deeper network structures, the sparsity at different levels of the network may exhibit different properties, making it a challenge to maintain sparsity without loss of accuracy. Gupta et al. (2024) proposed a novel, mechanics-inspired structured construction method. Similar to “Torque,” a force is applied during training to adjust convolutional layer weights around pivot points. This increases weight density near the pivot while promoting sparsity further away, enabling filter pruning with minimal information loss.

Structured pruning simplifies the model structure and improves the storage efficiency of the model by removing redundant convolutional kernels, while enhancing the corresponding ability of the model in the regions where signal changes are obvious, enabling ultrasound image analysis to be realized on portable ultrasound detectors. The structured pruning processed model can focus its performance on capturing the periodic fluctuation characteristics of the time domain signal (Ye et al., 2024), which significantly improves the sensitivity to signal changes and classification efficiency.

### 2.2 Knowledge distillation

Knowledge Distillation (Hinton, 2015) is an another model compression technique whose goal is to transfer knowledge from a larger, better performing ‘teacher model’ to a smaller ‘student model’, thus allowing the student model to achieve performance close to that of the teacher model with fewer computational resources and memory usage.

**FIGURE 5:**
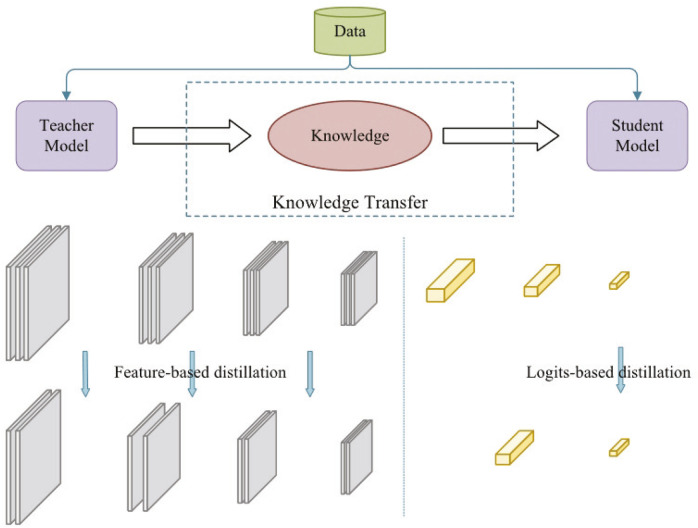
Knowledge distillation.

Knowledge distillation can reduce the size of the model regardless of the structural differences between the teacher and student models. When training the student model, the softmax output probability distribution of the teacher model is used as the training target, and a method is proposed to control the output probability distribution with a ‘temperature’ parameter, which can make the target ‘soft’. Given the logits z of the network, the category probability p of an image is calculated as Equation 1.

$$\left(z_{i},T\right)=\frac{\exp\left(z_{i}/T\right)}{\sum_{j}\exp\left(z_{j}/T\right)}$$ (1)

where T is the temperature parameter. When T = 1, the standard softmax function is obtained. As T increases, the probability distribution produced by the softmax function becomes softer, thus providing more information. As shown in Figure 5, knowledge distillation can be categorized into logit-based distillation and feature-based distillation based on the location of the knowledge in the teacher model (Chen et al., 2024).

Knowledge distillation can refine important timing features in the ultrasound signal. For example, in heart valve motion signals, the teacher model can extract the key time points of the waveform and guide the student model for feature extraction with low computational complexity. Meanwhile, knowledge distillation can obtain dynamic patterns of ultrasound signal patterns. Ren et al. (2023) used knowledge distillation to compress the laws of ultrasound signal waveform changes in the teacher model into feature representations in the student model for analyzing real-time cardiovascular signals. It has been shown that a deep denoising model incorporating convolutional neural networks can effectively mitigate such interference while maintaining key features in the signal (Micucci and Iula, 2022). Through distillation, the student model can learn the noise-resistant properties of the teacher model and improve the robustness of ultrasound signal classification. However, if the teacher model itself is sensitive to noise, it can be combined with adversarial training to enhance the robustness of the student model to noise.

#### 2.2.1 Logit-based distillation

In logit-based distillation, the student model learns the final logits of the teacher model output layer, which are the global representation. Since only the outputs need to be learned, the student model and the teacher model can have different architectures. Even if the student model is much smaller than the teacher model, it can still get good performance by imitating the output. Romero et al. (2014) and Mishra and Marr (2017) both verified that knowledge distillation can effectively improve deep network training, especially for the student model, which is shallow in depth and low in accuracy, and can learn more and more detailed features from the deep teacher model.

The attention mechanism also plays a key role in the development of knowledge distillation. Zagoruyko and Komodakis (2016) proposed a method for applying attention mechanism in the knowledge distillation process, called attention transfer. This approach extracts attention graphs from specific layers of the teacher model as a bridge for transferring knowledge between the teacher model and the student model, which allows the student model to fully learn the hierarchical information within the model. Jin et al. (2023) integrated the attention mechanism by aligning logits at the instance, batch and category levels, focusing on different levels of important features, and optimizing the knowledge transfer process. This enables the model to capture detailed features and transfer information more effectively, especially in complex ultrasonic signal processing tasks.

Focusing on “Neural Collapse” (Papyan et al., 2020), a phenomenon that refers to a series of geometric patterns that appear when a deep neural network approaches zero training error in an image classification task, Papyan et al. proposed a new perspective for optimizing knowledge distillation. Neural collapse simplifies the teacher-student learning process, allowing smaller student models to capture the key structures of the teacher model more easily.

Current logit-based distillation, because of the conflict between standard distillation loss and cross-entropy loss, leads to incorrect predictions even by highly accurate teacher models. Sun et al. (2024) introduced “refund logit-based distillation” to address the limitations of the current logit-based distillation. It is also effective in suppressing overfitting and eliminating potential misinformation from the teacher model while maintaining class relevance, ultimately allowing the student model to gain more valuable knowledge.

Zhao et al. (2022) proposed an improved knowledge distillation algorithm, Decoupled Knowledge Distillation (DKD), which decouples the loss of distillation into two parts: target category knowledge distillation (TCKD) and non-target category knowledge distillation (NCKD). The former focuses on the target categories and conveys the prediction results of the teacher model for the correct categories; the latter focuses on the probability distribution among the non-target categories and preserves the inter-class relationships.

Logits-based distillation enables the student model to learn the discriminative method of fuzzy samples more accurately by transferring the teacher model’s confidence difference in the classified samples, which can realize the effective recognition of weak features (Zhao et al., 2022) in ultrasound image signals, such as low-contrast lesions. In multitask classification, logit-based distillation can convey the recognition ability of the teacher model for complex signals and improve the adaptability of the student model for dynamic signals.

#### 2.2.2 Feature-based distillation

Feature-based distillation learns feature representations of data samples at different levels, focusing on the local perceptual ability of the model and the expressive ability of the middle layer. Feature distillation can outperform logit-based distillation but is relatively complex to implement and requires additional computation and memory consumption to refine deep features during training (Heo et al., 2019).

Optimization for feature-based distillation often starts with the structure of the teacher-student models. Since the mapping of deep neural network models from the input space to the output space needs to go through many layers, Yim et al. (2017) defined the knowledge to be transmitted by the information flow of features between layers, which is obtained by calculating the inner product between the features of the two layers. Huang et al. (2023) used neuron selectivity to align selectivity patterns between teacher and student models, enhancing student network performance. They also introduce a feature-based distillation strategy, including multi-scale feature distillation, which overcomes single-scale limitations, and self-mutual information distillation, combining self-supervision in the student model with mutual supervision from the teacher. (Heo et al., 2019) placed the distillation location before the ReLU activation function of the neural network, which eliminated the redundant information that would adversely affect model compression and allowed the student network to learn more effective information from the teacher network. Chen et al. (2022) proposed to improve the feature distillation by using a projector ensemble (projector ensemble) to improve the feature distillation. Adding multiple projectors to the student model solves the mismatch between the teacher and student feature spaces and improves the performance of the image classification task.

Liu et al. (2024) proposed a large kernel attention network based on pyramid segmentation, using the dynamic feature distillation module can extract the features of different layers, effectively improved the performance of the image super-resolution model. Tian et al. (2019) developed a novel distillation technique by using the Contrastive Learning (Le-Khac et al., 2020) approach to develop a novel distillation technique that enables teacher and student models to project the same inputs onto adjacent representations and different inputs onto separated representations.

Feature-based distillation requires aligning different levels of feature representations between the teacher model and the student model, which leads to the alignment of the two in feature space becoming challenging. Also passing information about the middle level of the teacher model increases the training time and memory requirements of the model. Whereas the training cost of the logit-based distillation is lower, but the performance is not satisfactory compared to feature-based distillation. Therefore, both of them still need to be further optimized to reduce the problems of knowledge distillation in terms of complexity, computational cost and task suitability.

### 2.3 Low-rank decomposition

Most deep neural networks are over-parameterized and exhibit large computational overhead, making signal recognition inefficient. Low-rank decomposition Kolda and Bader (2009) refers to sparsifying the convolution kernel matrix by combining dimensions and imposing low-rank constraints. Since the weight vectors are mostly distributed in low-rank subspaces, the convolution kernel matrix can be reconstructed with a small number of basis vectors to achieve the purpose of reducing the storage space (Cheng et al., 2017). By approximate decomposition of the weight matrix or feature representation of the neural network, redundant information is removed and the model is made more compact. However, over-decomposition may weaken the ability to perceive the dynamic changes in time and have an impact on the temporal consistency of the ultrasound signal. By using an adaptive low-rank approximation method (Lu, 2024), the decomposition level is dynamically adjusted to avoid the loss of critical time series information.

Yin et al. (2022) proposed a budget-aware neural network compression method based on Tucker decomposition (Weber et al., 2025), called BATUDE. Maintain or improve model performance while meeting computational budget constraints through automated tensor rank selection and globally optimal rank learning strategies. The method not only simplifies the training process, but also enables the model to automatically learn features suitable for specific data, improving the effectiveness of feature extraction while providing a more favourable feature representation for subsequent image recognition tasks. Futhermore, Yadav et al. (2022) proposed an efficient neighbor search method based on matrix decomposition, which is optimized for cross-encoder models. Through matrix decomposition technology, the computational cost of neighbor search is significantly reduced while maintaining high retrieval accuracy. This method provides a new idea for solving the problem of efficient search in large-scale data sets. Low-rank decomposition can extract low-dimensional structures from high-dimensional data and reveal the interactions between different modalities.

The diversity and complexity of biomedical data require new data analysis methods. Low-rank decomposition, as a powerful model compression technology, is widely used in medical signal processing, such as image signal denoising, super-resolution reconstruction, feature extraction, etc. CP decomposition (Zhou et al., 2019) and Tucker decomposition are particularly prominent in image reconstruction and noise removal (Wu et al., 2018). Burch et al. (2025) systematically reviewed the applications of low-rank factorization in biomedical data analysis and explored the potential of quantum computing to address the challenges faced by traditional low-rank decomposition. Combined with quantum computing, tensor decomposition is expected to further promote the development of precision medicine, especially in terms of data scale and processing efficiency.

## 3 Operational optimization

With the increasing demand for ultrasound signal classification tasks in real-time and embedded devices, it is especially important to design optimization strategies that efficiently process ultrasound data and balance real-time and accuracy. The operational optimization technique focuses on considering how to enhance the extraction ability of key features and reduce redundant calculations. Convolution serves as the logical basis for the operation of the model, and the lightweight design of convolution can maximize the computational efficiency of the network, which is convenient for the model to be used on mobile devices such as portable ultrasound detectors.

In this chapter, lightweight optimization algorithms suitable for ultrasound signal classification, including decoupling and portability modules, will be explored in detail in light of the ultrasound signal characteristics, especially for the temporal dynamic characteristics of M-mode ultrasound signals and the frequency domain characteristics of time-domain signals, which will provide technical support for further advancing ultrasound diagnosis.

### 3.1 Decoupling

**FIGURE 6:**
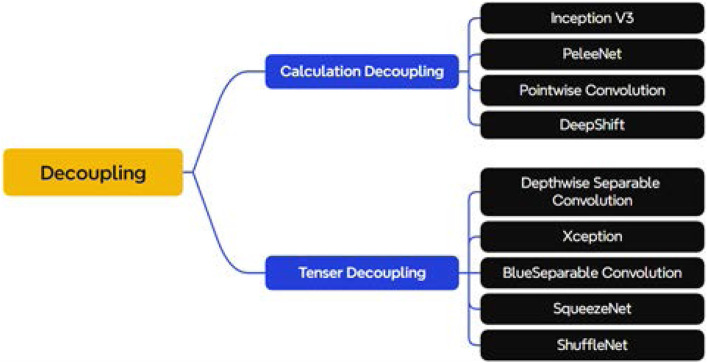
Classification of decoupling methods.

In convolutional neural networks, there are dependencies between modules, which can be as small as a certain weight or as large as the entire network layer. Therefore, the coupling degree can be utilized to define the dependency of modules in the model, and the lower the coupling degree, the lower the dependency between modules, and the greater the independence, reusability and portability of modules. Classical lightweight modules such as deeply separable convolution (Howard, 2017) and channel shuffling (Zhang et al., 2018) can be explained by the idea of decoupling. As shown in Figure 6, this section will divide some manually designed convolutional structures with the concept of decoupling, which can be mainly categorized into two parts: calculation decoupling and tensor decoupling.

#### 3.1.1 Calculation decoupling

The size of the convolution kernel determines the extent of the sensory field on the input image. The larger the convolution kernel, the larger the perception field and the better the feature extraction effect. In order to ensure the classification effect, most early neural network models, such as AlexNet Krizhevsky et al. (2012), used large convolution kernels for feature extraction.

**FIGURE 7:**
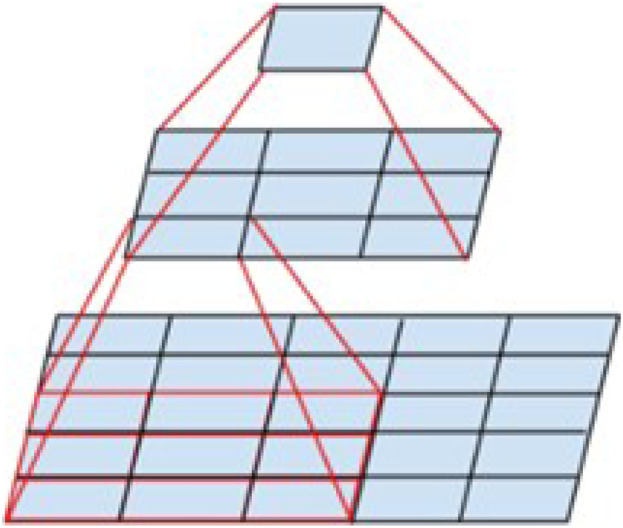
The 5*5 convolution is replaced by a smaller one.

However, large convolutional kernels significantly increase computation and memory consumption for processing high-resolution images such as medical images, leading to a decrease in the efficiency of the model during training and testing, at the same time, larger convolutional kernels imply a wider sensory field, which can mishandle important local features in the ultrasound images. Researchers have therefore explored convolution kernel sizing. Replacing a large-size convolutional kernel with multiple small-size convolutional kernels can be referred to as calculation decoupling because it changes the way the original convolution operates. This idea originates from Inception V3 proposed by Szegedy et al. (2015). As shown in Figure 7, the 5*5 convolution was replaced with a multilayer network with fewer parameters: the first layer is a 3*3 convolution, and the second layer is a fully connected layer on the first 3*3 output grid. The replacement reduces the number of model parameters by 27.8% and the sensory field is unchanged before and after the split.

**FIGURE 8:**
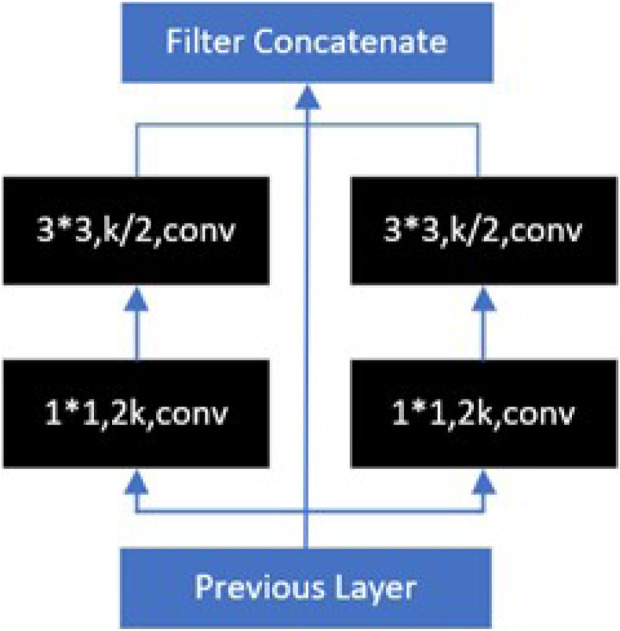
2-way dense layer in PeleeNet.

Wang et al. (2018) were inspired by the Inception model and proposed the PeleeNet model. PeleeNet introduces a 2-way dense layer structure, as shown in Figure 8, which is a parallel structure of multi-scale convolutional kernels (e.g., 1*1, 3*3), and fuses different scales of sensory fields within a single dense layer, which effectively enhances the ability to capture features of different scales. In the eight-neighborhood pixel, the 3*3 convolution is the smallest odd convolution kernel size that can capture the features, so various lightweight models often use the 3*3 convolution for the convolution splitting operation to reduce the computation amount of the model.

In addition to using 3*3 regular convolutions to reduce the number of network parameters, 1*1 convolutions can be used to perform dimension upscaling and dimension downscaling operations on feature maps to achieve the same purpose. Point-by-point convolution (Hua et al., 2018) computes a linear combination of the output of the depth convolution by 1*1 convolution to obtain a new feature.

Although there are differences in the design structure of the above decoupling strategies, their essence is realized by reducing the size of the number of operations in the multiplication process, which mathematically disassembles the standard single multiplication operation and replaces some of the multiplication operations with addition operations to achieve the effect of improving the speed of the model operation.

The DeepShift (Elhoushi et al., 2021) replaces the floating-point multiplication operation in the original forward propagation by performing shift-by-bit and inverse-by-bit operations to reduce the computation time required in the model inference process. Shift and inverse operations are faster in hardware circuit devices, so using them to replace the product operation can speed up the model. DeepShift’s design idea is novel, using the underlying algorithm to thoroughly accelerate the convolution from the perspective of accelerating hardware resources.

At this stage, the model design approach for convolutional operations relies heavily on the design of the underlying hardware devices, and the binary arithmetic process will make the power operation unavoidable errors in the substitution process, which will lead to the model being limited in practical applications. However, this kind of operation substitution can still be used as a direction for future research.

#### 3.1.2 Tensor decoupling

Unlike the calculation decoupling strategy that reduces the amount of computation by replacing the large-size convolution with a small-size convolution, the tensor decoupling operation chooses to disassemble the conventional convolution in the spatial dimension and the channel dimension and performs it in steps, separating the variables or features that are originally closely connected, so that certain parts or parameters in the model can be varied independently, thus reducing the interdependence between them.

**FIGURE 9:**
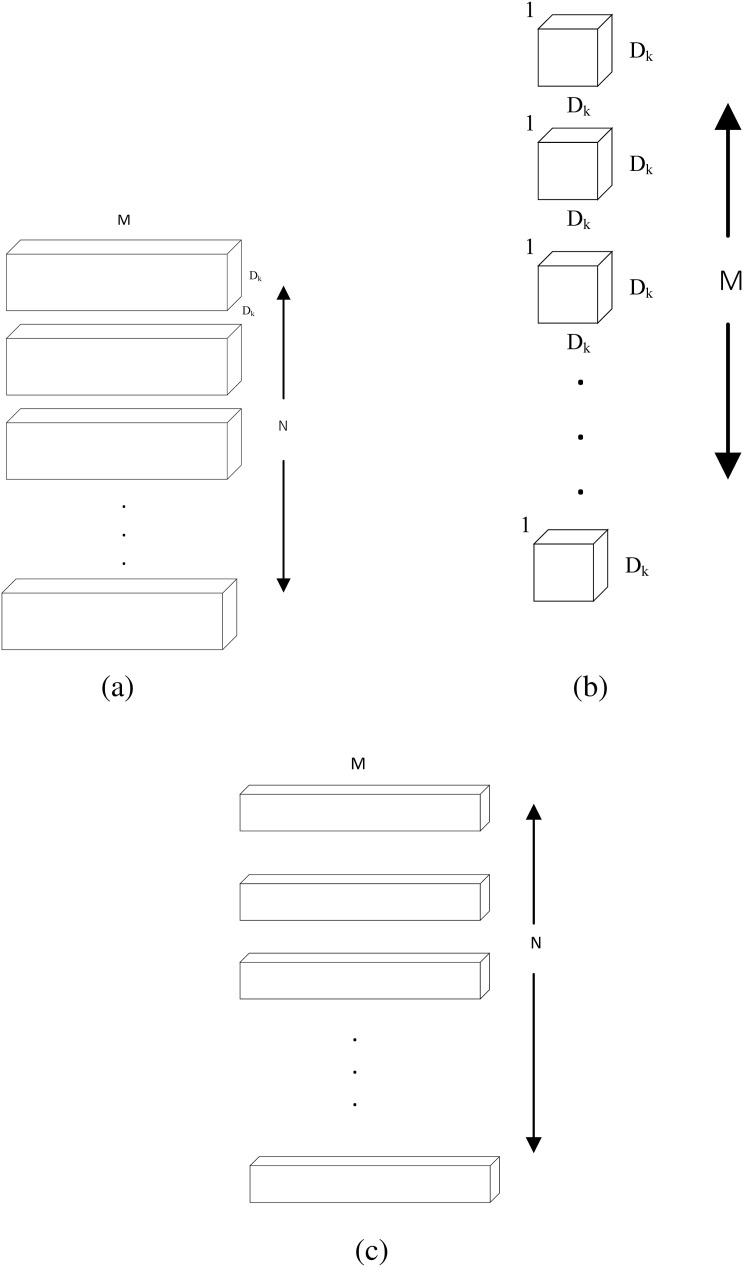
Depthwise separable convolution. **(a)** Standard Convolution Filters. **(b)** Depthwise Convolution Filters **(c)** Pointwise Convolution Filters.

The design of conventional depth-separable convolution belongs to the category of tensor decoupling, as shown in Figure 9, depth-separable convolution Howard (2017) decomposes the conventional 3D convolution into a depth convolution in two-dimensional space and a point-by-point convolution that modifies the number of channels to turn the 3D input features into independent 2D planar features and channel dimensions. Deep convolution extracts local spatial features in each channel and combines them with point-by-point convolution to complete feature fusion. The decoupling in depth-separable convolution reduces computational complexity and number of parameters, making DS Conv particularly suitable for environments with limited computational resources, such as portable ultrasound devices. The depth-separable convolution can also be combined with an adaptive filter (You and Crebbin, 2022) for removing scattering noise from ultrasound images to improve the quality of ultrasound signals.

However, traditional design methods do not consider the interrelationships of features within a convolutional kernel, which leads to limited performance of the model in complex feature learning. To address this limitation, Li et al. (2022) proposed Blueprint Separable Convolution (BSConv), focusing on decoupling and recombination within the convolutional kernel. BSConv separates and recombines features efficiently by introducing a different feature separation strategy, which explicitly takes into account the interactions of features within the convolutional kernel.

The Fire Module in SqueezeNet Iandola (2016) is also a decoupled calculation, the Fire Module consists of the squeeze layer and the expand layer. The “compression-expansion” operation in this design decouples spatial features (3*3 convolution) and channel features (1*1 convolution), allowing the model to separately model inter-channel and spatial domain features without having to use a single standard convolution operation to process all features simultaneously.

While the decoupling operations in the above models all disassemble the N*N convolution into a combination of n*n convolutions, in InceptionV3 Szegedy et al. (2016) the authors propose to disassemble a square convolution of one (7*7) into a stack of 1*7 and 7*1. This decoupling operation reduces the 49 multiplication operations to 7.

Features belonging to different channels after decoupling cannot be transferred. The channel shuffling operation in ShuffleNet (Zhang et al., 2018) disrupts the feature map channels so that features originally belonging to different channels can be mixed together in the subsequent convolution operation, thus realizing the exchange of information between different channels.

### 3.2 Portability modules

Convolutional modules are artificially constructed application programming interfaces (APIs) that can be invoked directly in programming using abbreviations, and wrapper packages usually contain fixed convolutional structure modules for high portability. In this section, several lightweight portable modules that can be applied to ultrasound image classification are systematically described.

#### 3.2.1 Residual module

**FIGURE 10:**
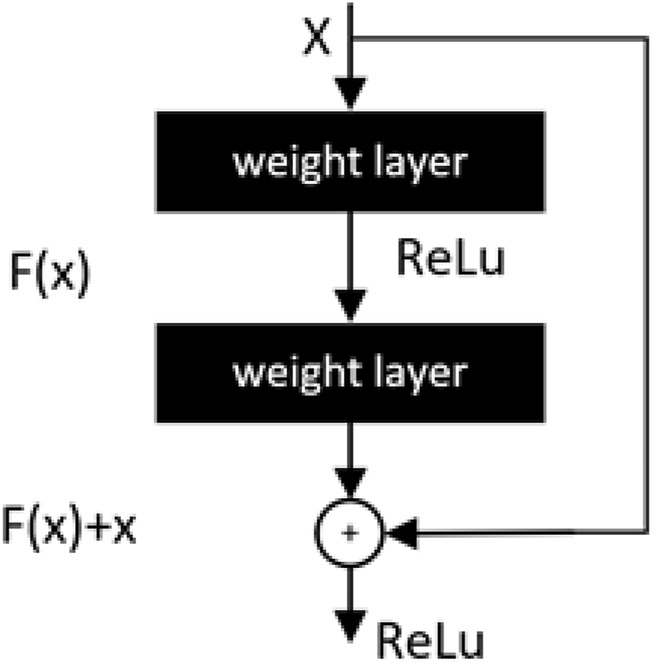
Basic residual module.

The residual module proposed by He et al. (2016) is a key part of the advancement of lightweight development of convolutional neural networks. The deeply separable convolution in MobileNetV1 (Howard, 2017) and the inverse residual in MobileNetV2 (Sandler et al., 2018) draw on the principle of the residual module. In mathematical statistics, residuals are usually used to represent the difference between the actual observed value and the fitted value. As shown in Figure 10, in the structure of a neural network a stacked layer, when the input is x, the learned feature is the mapping H(x). Compared with the original feature H(x), the difference is easier to learn directly, so the residuals as the difference computes the learned feature can be expressed as: F(x) = H(x)-x and the original mapping is reshaped as F(x)+x.

From the above equation, when the residual F(x) is 0, the stacking layer only does constant mapping and the network performance should remain unchanged. But in fact, the residuals are not 0, thus causing the stacking layer to constantly learn new features on top of the input features and thus the network will have better performance (Chollet, 2017).

**FIGURE 11:**
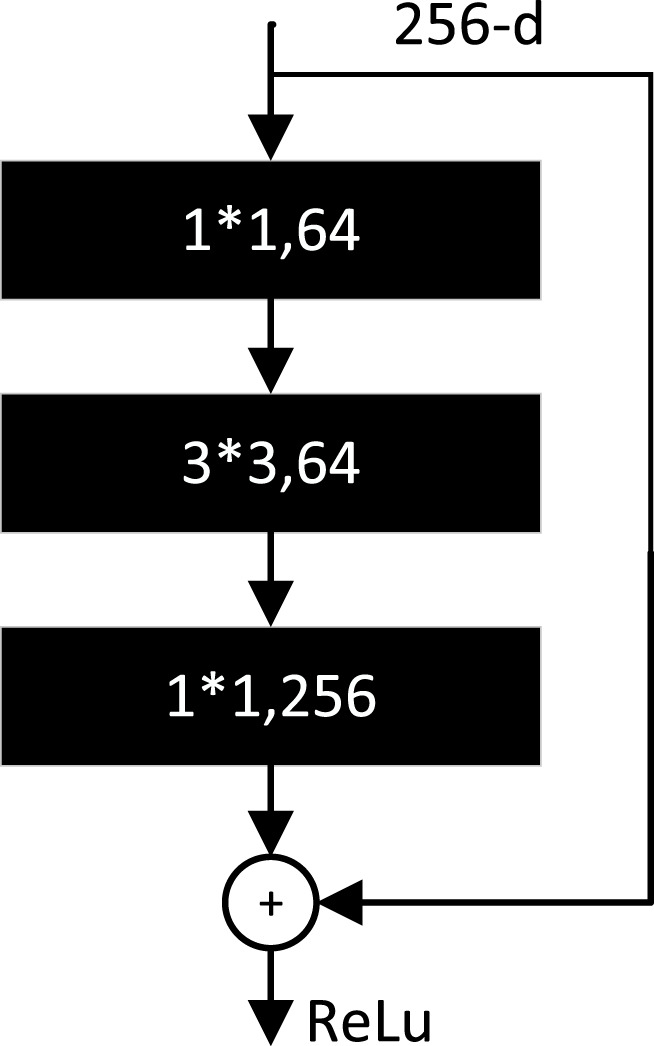
Bottleneck residual module.

The BottleNeck residual module is the basic unit of ResNet (Han et al. 2015). It consists of three convolutional layers as shown in Figure 11. The number of channels at input is restored by reducing the dimensionality using 1*1 convolution, then using 3*3 convolution for feature extraction, and finally using 1*1 convolution to raise the dimensionality.

**FIGURE 12:**
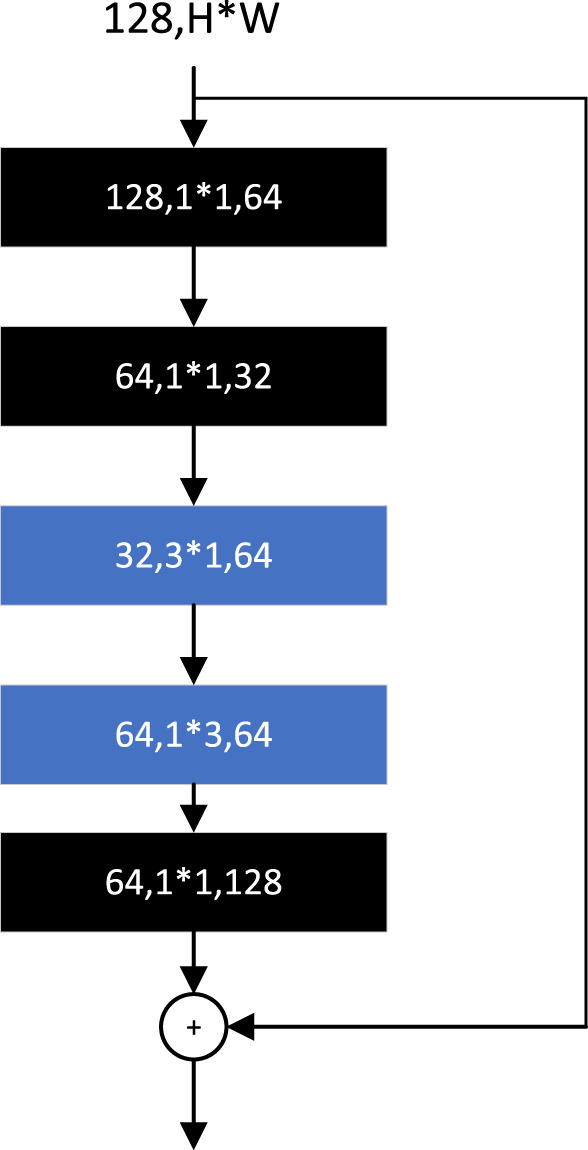
SqueezeNext module.

The basic module of SqueezeNext (Gholami et al., 2018) is adapted from BottleNeck. The 3*3 convolution is factorized into the sum of two 2D convolution operations, 3*1 and 1*3, as shown in Figure 12, and a set of 1*1 convolutions is added before the start of the BottleNeck structure, which is used to reduce the number of channels. By adjusting the number of 1*1 convolutions, the number of channels in each layer of convolution can be flexibly controlled to ensure that the two convolution operations obtained from the 3*3 convolution factorization are always in the lower dimensional channels. The final 1*1 convolution in the module is used to restore the features to the same dimension as the input channels.

**FIGURE 13:**
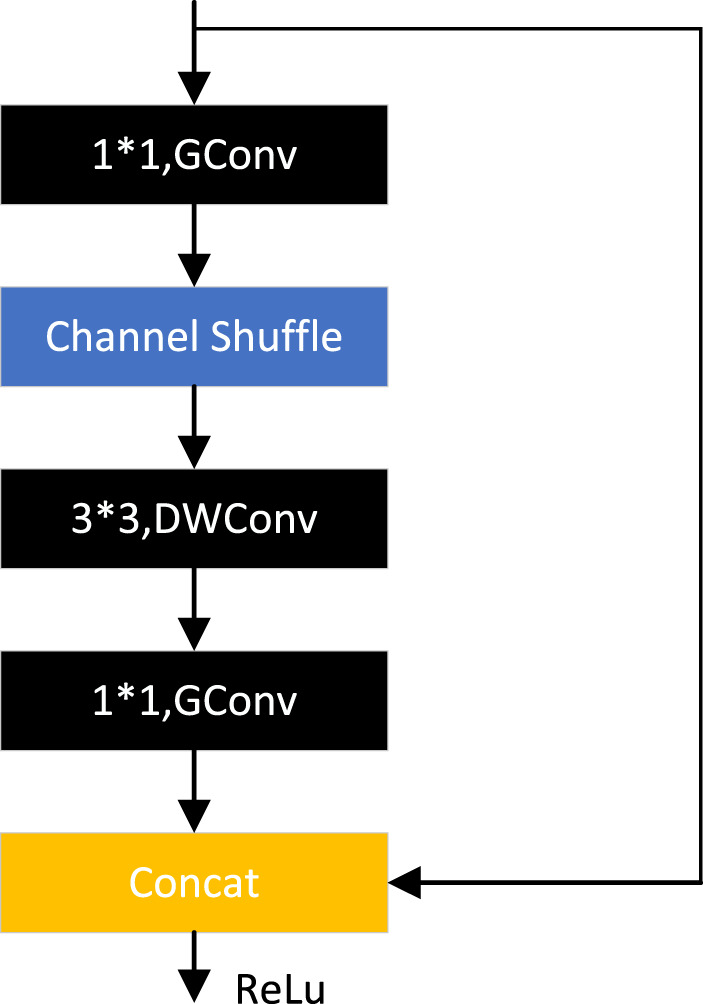
ShuffleNet module.

ShuffleNetV1 (Zhang et al., 2018) is optimized based on the bottleneck structure, as shown in Figure 13, replacing the normal 3*3 convolution with a 3*3 DW convolution, replacing the 1*1 convolution with a 1*1 grouping convolution, and adding a channel cleaning operation after the first 1*1 grouping convolution. Then the original element sum operation is converted to channel cascade concat. 3*3 DW convolution can significantly reduce the number of parameters, but when the number of channels is too high using 1*1 convolution many times will increase the amount of computation. Group convolution perfectly solves this problem, after the experimental demonstration, 0.25 times the number of groups tend to sustain better results, which indicates that wider feature maps can bring better results for smaller models.

The spatial bottleneck module of DetNet (Li et al., 2018) is also an improvement of the bottleneck structure, which replaces the original 3*3 convolution with the cavity convolution, allowing the feeling field to be expanded arbitrarily without introducing additional parameters. The spatial bottleneck module can greatly improve the ability of the model to localize segmentation, and at the same time obtain the very important multi-scale information in the vision task. For ultrasound images that are susceptible to noise, the spatial bottleneck module allows for better feature extraction.

**FIGURE 14:**
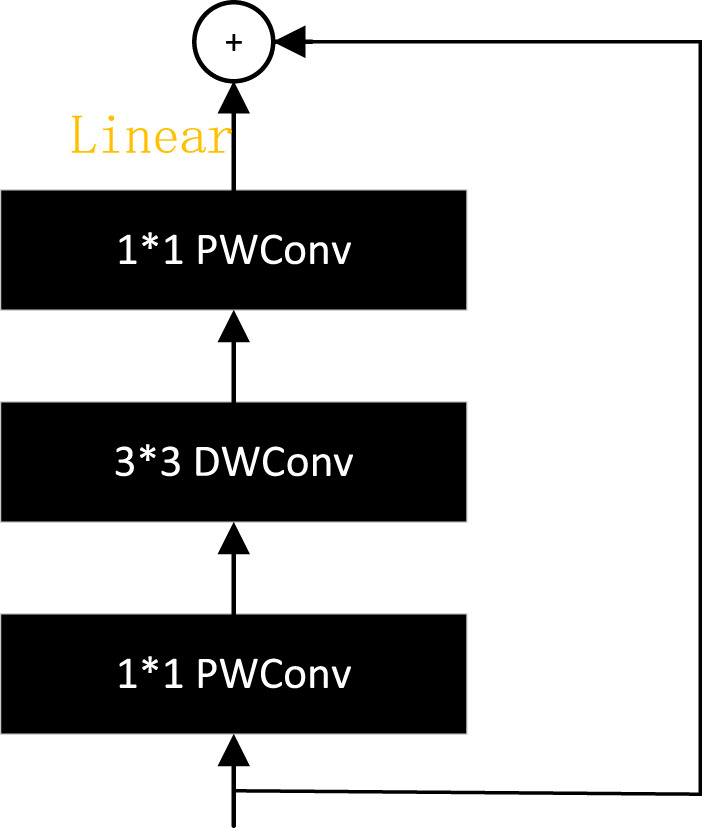
Inverse residual module.

MobileNetV2 Sandler et al. (2018) proposes a reverse residual module, as shown in Figure 14, which first lowers the dimensions, then raises them, and replaces the ReLU activation with a linear activation. The inverse residual module has both the optimization characteristics of the bottleneck structure and disassembles the convolution through the depth separable convolution, using a lighter weight convolution to further reduce the model computation. The ghost bottleneck with a stride of two in GhostNet is a standard residual module structure. A depth-separated convolution module is added between the two ghost modules to reduce the amount of computation (Han et al., 2020).

Khan et al. (2024) proposed ESDMR-Net, a deep convolutional network architecture with Squeeze-Excitation (SE) module, to better handle high-frequency information and feature variations in bruise images. The SE module extends the multi-scale information through deep separable convolution and extracts compact salient information through bottleneck layers to enhance the feature representation capability. The deep structure of the network enables feature refinement and accumulation through multi-branching design and reuse of SE modules, making it more robust in dealing with highly variable features.

Residual modules are widely used in building lightweight neural networks, often replacing standard convolutions with more lightweight depth-separable convolutions to further reduce computation on the basis of optimized structure (Qin et al., 2024). However, the trade-off is that the embedding of the residual module will make the original model structure relatively complex, and it is often necessary to utilize model compression to further reduce the memory footprint of the model.

#### 3.2.2 Grouped convolution

Originating in 2012, AlexNet (Krizhevsky et al., 2012) split the convolution, and grouped convolution effectively reduced the computational complexity (FLOPs) and achieved structured sparsity. When the number of groups is equal to the number of input channels, grouped convolution can be transformed into a DW convolution to further reduce parameters. Each branch of the ResNeXt module (Xie et al., 2017) employed an identical convolutional topology. The idea of grouped convolution is actually to adjust the number of channels involved in the convolution operation. By splitting the number of channels for convolution, each grouping is executed in parallel. The number of subgroups depends on the hardware resource configuration currently in use and is related to the design of the network structure being embraced. The design of grouped convolution is more in line with GPU hardware design principles and thus runs faster than the Inception (He et al., 2016) module with manually designed convolutional details.

Grouped convolution allows more channels to be used with fixed FLOPs and increases the capacity of the network, so networks with grouped convolution (Howard, 2017; Zhang et al., 2018; Xie et al., 2017; Ma et al., 2018; Guo et al., 2022) can maintain high accuracy while reducing FLOPs. However, increasing the number of channels leads to higher memory access costs (MACs). ShuffleNetV2 (Ma et al., 2018) introduces a channel splitting module in its basic unit, which functions similarly to grouped convolution but helps mitigate the excessive MACs associated with an increased number of grouped convolutions.

The Ghost module in GhostNet (Han et al., 2020) divides the results generated by convolution into two groups. One group retains part of the original convolution, and the other group is optimized for the redundant features in the output that are similar to each other. A small number of base features are first generated from some of the standard convolutional layers. Secondly, a series of linear transformations are used to further generate new features, which are called Ghost feature maps. A large number of similar redundant feature maps generated by the convolution operation are replaced by Ghost features, achieving model speedup.

Grouped convolution allows different groups of channels to focus on different types of features, separating local features from global features. For example, certain groups focus on edge information, while others focus on texture or shape, avoiding interference from scattered noise in all channels.

#### 3.2.3 SE module

SE module (Squeeze-Excitation) (Hu et al., 2018) includes two operations: Squeeze and Excitation. It is hoped that the model can autonomously learn the dependencies between different channels and obtain the relationships between features. Squeeze uses a 1*1 global average pooling operation to compress the spatial features of each channel into a scalar to obtain a global description of the channel. Excitation performs dynamic adaptive adjustment of the channel weights. The global description generated in the Squeeze part is fed into a sub-network with two fully connected layers for learning the dependencies between the channels. The global description obtained in the Squeeze part amplifies the sensory range, avoiding the limitation that small sensory fields in the shallow network are unable to sense more features. The Excitation part is an automated gating mechanism that allows the model to adaptively focus on the important feature channels.

The SE module is portable and can provide significant performance improvements for deeper architectures with minimal additional computational cost. The SE module can be embedded in the ResNet residual network (He et al., 2016), where it is placed after the output of the main branch of each residual module, and the weighted outputs are obtained after Squeeze and Excitation. The main branch features processed by the SE module are then added with the residual branches to obtain the final output features. In MobileNetV3 (Howard et al., 2019), the SE module is added after the DW convolution block, further improving accuracy without increasing time loss.

The embedding of the SE module can help the model to better learn the information of each channel and enhance the network’s ability to pay attention to the key features when recognizing the dynamically changing time-domain information of ultrasound signals and improve the robustness of the anomalous signal detection. Jiang et al. (2019) combined the residual module and the Squeeze-and-Excitation module to design a small SE-ResNet module for the classification of breast cancer histopathology images to reduce the training parameters of the model and the risk of over-fitting. Zhang et al. (2020) used a similar approach to solve the problem that the model cannot extract accurate features of long-term sequences in the task of signal classification.

#### 3.2.4 Attention mechanisms module

Attention Mechanism Modules (Guo et al., 2022) are lightweight and generalized modules that allow feature focusing in the channel dimension and spatial dimension, and integration of independent dimensions of both. Thus,the attention mechanism module can be categorized into channel attention module (CAM) and spatial attention module (SAM). The attention mechanism module is similar to the SE module, both CAM and SAM use a double-pooling operation, that is, adding a global maximum pooling operation on top of the global average pooling. This dual-pool operation structure can extract richer high-level feature information. The encapsulated attention mechanism module can be directly embedded behind the regular convolutional layer of the feedforward neural network without any additional computational overhead.

The Coordinate Attention (CA) module (Hou et al., 2021) generates two 1D feature representations by aggregating the global information of the input feature map in the height and width directions, respectively. Unlike traditional attention mechanisms, the CA module captures the dependencies of features in both vertical and horizontal directions, making it easier for the model to capture the exact location of the target. Therefore, embedding the CA module in the model that performs ultrasound signal classification can help the model more accurately identify the location of the disease and improve the accuracy of diagnosis (Ansari et al., 2024).

MobileVit (Liu et al., 2017) integrates the Transformer module into a lightweight convolutional neural network, which retains the efficient local feature extraction capability of the network and enhances the capture of global relationships. By combining the advantages of both, MobileVit can be adapted to multitasking scenarios, and its efficient global feature capture capability can be used in complex scenarios such as ultrasound signal processing.

Denoising of ultrasound images may result in the loss of local detail features. SBCFormer, proposed by Lu et al. (2024), solved this problem by designing a two-stream block structure. One stream is used to reduce the feature map size and apply the attention mechanism, while the other stream is used as a “pass-through channel” to retain the local information of the input feature map.

### 3.3 Analysis and summary

This subsection summarises the lightweight convolutional neural networks. Table 2 demonstrates the performance of the lightweight models on the ImageNet dataset. In order to fix a benchmark for comparison, these models are evaluated on the ImageNet dataset, using the parametric counts, FLOPs, and MAC metrics to measure the lightweight effect and also focusing on the classification accuracy of the models. Analogising the performance of these models on the ImageNet dataset can provide ideas for the ultrasound signal analysis task. FLOPs and a number of covariates metrics do not fully reflect the actual efficiency of the models (Lai et al., 2018) and still need to be optimised according to the task scenario. Table 3 demonstrates the lightweight techniques used in these models, and it is easy to see that a combination of optimisation techniques is often required to achieve a model that maintains higher accuracy while improving computational efficiency.

**TABLE 2:** The comparison of lightweight models on ImageNet.

Model	Parameter(M)	FLOPs(M)	MACs(G)	TOP-1 ACC. (%)	TOP-2 ACC. (%)
SqueezeNet (2016)	4.8	0.82	0.35	57.5	80.3
InceptionV3 (2016)	23.62	5.72×1024	5.72	77.9	93.7
MobileNetV1 (2017)	4.2	575	0.55	70.6	89.5
Xception (2017)	22.85	8.4×1024	8.42	78.8	94.3
SqueezeNeXt (2018)	3.2	708	0.69	67.5	88.2
MobileNetV2 (2018)	3.4	300	0.29	72.0	91.0
ShuffleNet (2018)	3.46	140	0.14	72.6	—
ShuffleNetV2 (2018)	2.3	146	0.15	71.8	—
PeleeNet (2018)	2.8	508	0.51	72.6	90.6
MobileNetV3 (2019)	3.2	265	0.21	75.2	—
EfficientNetV1 (2019)	5.3	390	0.18	77.1	93.3
GhostNet (2020)	5.2	141	0.14	73.9	94.1
EfficientNetV2 (2021)	24	280	0.28	83.9	—
VanillaNet (2023)	15.5	520	0.52	72.49	79.66
MFDNet (2023)	11.46	384	0.38	77.8	—
SBCFormer (2024)	5.6	700	0.7	75.8	—
MobileNetV4 (2024)	9.2	220	0.22	82.9	—

**TABLE 3:** Models and their lightweight technologies.

Model	LightWeight technology
SqueezeNet (2016)	Calculation decoupling
InceptionV3 (2016)	Calculation decoupling
MobileNetV1 (2017)	Tensor decoupling (Depthwise separable convolution)
Xception (2017)	Tensor decoupling (Depthwise separable convolution)
SqueezeNeXt (2018)	Tensor decoupling (Depthwise separable convolution); Residual module
MobileNetV2 (2018)	Tensor decoupling (Depthwise separable convolution); Residual module (Inverted residuals)
ShuffleNet (2018)	Group convolution; Channel shuffle
ShuffleNetV2 (2018)	Group convolution; Residual module (Inverted residuals)
PeleeNet (2018)	Calculation decoupling; Tensor decoupling (Depthwise separable convolution)
MobileNetV3 (2019)	SE module; Tensor decoupling (Depthwise separable convolution)
GhostNet (2020)	Tensor decoupling (Depthwise separable convolution)
EfficientNetV2 (2021)	Tensor decoupling (Depthwise separable convolution)
VanillaNet (2023)	SE module; Attention mechanisms module
MFDNet (2023)	Residual module; Attention mechanisms module
SBCFormer (2024)	Residual module (Inverted residuals); Attention mechanisms module
MobileNetV4 (2024)	Attention mechanisms module

## 4 Future research directions

Based on the above analysis, the current lightweight technology has limitations in its wide application to a certain extent. Future research should focus on the following promising directions:

### 4.1 Data imbalance

Designing a lightweight model for ultrasound signals requires overcoming the impact of experimental data imbalance on the model (Mone et al., 2023). Multiple analysis methods can be used to reduce the bias that may be caused by a single experimental method. For lightweight models, methods such as adjusting the loss function and category weights can be used to give a larger weight to minority categories, thereby reducing the impact of data imbalance on model performance and improving the model’s ability to identify different categories.

### 4.2 Lack of statistically significant clinical efficacy

Existing AI-assisted diagnosis and treatment methods are basically still in the research stage, lacking extensive clinical trials, and there is a gap between translational research and clinical application (Dhage et al., 2021). In the future, large-scale randomized controlled trials (RCTs) can be conducted to evaluate the clinical effectiveness of the model. Additionally, different organs and tissues have different ultrasonic features, such as periodic heartbeat signals, non-linear muscle tissue echoes, and time domain features depend on dynamic changes within the time window. Therefore more extensive evaluation across diverse ultrasound datasets, incorporating different probes, imaging frequencies, and tissue types, is necessary to validate the generalizability of these models. Multi-center studies and cross-dataset validation will be critical to ensuring robust performance in varied clinical settings.

### 4.3 Interpretability and generalizability of the model

The interpretability of model research requires sufficient theoretical guidance and experimental analysis. Many studies are based on small-scale data sets or data from specific institutions and lack multicenter, multiethnic, and multienvironmental data validation (Ansari et al., 2024). More ablation experiments are necessary to focus more on which part of the lightweight CNN leads to performance improvement. This is a key factor to accurately improve image recognition performance, rather than blindly stacking techniques to improve performance (Chandrasekar et al., 2022). Future research should focus on optimizing lightweight CNNs for real-time ultrasound applications.

## 5 Conclusion

This paper presents a detailed review of the current state of research and the challenges of lightweight techniques for the task of ultrasound signal classification. Pruning and knowledge distillation techniques improve diagnostic accuracy while reducing model complexity, especially structured pruning that removes redundant filters in M-mode ultrasound and focuses on critical temporal features of the time-domain signal. Operational optimization techniques optimize computational efficiency while improving feature extraction capabilities, adapting to the deployment of embedded devices. In addition, for the scattering noise and acoustic shadow effect in ultrasound signals, adding feature enhancement modules to the network can effectively improve the robustness and classification accuracy of the model. In the future, based on the lightweight model architecture, we will use model compression techniques to design model architectures specifically for M-mode ultrasound and time series signals, which will further promote their application in diverse clinical scenarios such as fetal health detection and lung disease diagnosis. Through continuous research and innovation, lightweight technology will become an important bridge connecting AI with the practical needs of healthcare.
